# Simultaneous Determination of Six *Uncaria* Alkaloids in Mouse Blood by UPLC–MS/MS and Its Application in Pharmacokinetics and Bioavailability

**DOI:** 10.1155/2020/1030269

**Published:** 2020-08-17

**Authors:** Lianguo Chen, Jianshe Ma, Xianqin Wang, Meiling Zhang

**Affiliations:** ^1^The Third Clinical Institute Affiliated to Wenzhou Medical University, Wenzhou People's Hospital, Wenzhou 325000, China; ^2^School of Basic Medicine, Wenzhou Medical University, Wenzhou 325035, China; ^3^Analytical and Testing Center of Wenzhou Medical University, Wenzhou 325035, China

## Abstract

A specific ultraperformance liquid chromatography-tandem mass spectrometry (UPLC-MS/MS) method has been developed for the simultaneous determination of six *Uncaria* alkaloids in mouse blood with midazolam as the internal standard (IS). Only 20 *μ*L blood was needed for sample preparation, and the protein was precipitated with acetonitrile. The UPLC BEH C18 column (2.1 mm × 100 mm, 1.7 *μ*m) was used for chromatographic separation. The mobile phase consisted of 0.1% formic acid and acetonitrile with gradient elution within 5.5 min. Multiple reaction monitoring (MRM) and the positive electrospray ionization model were used for quantitative analysis. The accuracy of the UPLC-MS/MS method ranged from 86.5% to 110.4%. The precision for intraday and interday was ≤15% each. The mean recovery and the matrix effects were found to be 64.4-86.8% and 94.1-109.4%, respectively. The calibration curves in blood were linear in the range of 1-1000 ng/mL with a favorable correlation coefficient (*r*^2^) of 0.995. The pharmacokinetic results showed that six *Uncaria* alkaloids metabolized rapidly in mice with a half-life between 0.6 h and 4.4 h. The bioavailability of corynoxeine, isocorynoxeine, rhynchophylline, isorhynchophylline, hirsutine, and hirsuteine was 27.3%, 32.7%, 49.4%, 29.5%, 68.9%, and 51.0%, respectively, which showed satisfactory oral absorption of each alkaloid.

## 1. Introduction

*Uncaria* is a dry hooked branch of Rubiaceae, and it is a traditional Chinese medicine. It has been included in the Chinese Pharmacopoeia; for example, *Uncaria rhynchophylla.* (*Miq.*) *Miq. ExHvail*., *Uncaria sinensis (Oliv.) Havil*., *Uncaria macrophylla Wall.*, *Uncaria sessilifructus Roxb*., and *Uncaria hirsuta Havil*. [1, 2]. Among them, Hook Vine (*Uncaria rhynchophylla.* (*Miq.*) *Miq. ExHvail*.) has been widely studied and is commonly used [3, 4].

The main components of Hook Vine include alkaloids, flavones, phenols, coumarins, organic acids, quinol nosides, and triterpenoids; however, research on the pharmacodynamic sites has been focused on alkaloids [1]. *Uncaria* alkaloids are mainly divided into two types (oxidized indole alkaloids and nonoxidized indole alkaloids) based on whether C-2 is oxidized or not [5–8]. The alkaloid content in Hook Vine varies greatly due to the variety, geographical environment, rain, and harvest season. Even if it is the same medicinal material, the alkaloid content varies greatly because of the different parts (the part of the hooked stem branches are the most). So far, close to a hundred alkaloids have been identified in Hook Vine, among which the main indole alkaloids include rhynchophylline, isorhynchophylline, corynoxeine, and isocorynoxeine, and the main nonoxidized indole alkaloids include hirsutine and hirsuteine. Modern pharmacological studies have shown that *Uncaria* alkaloids have antihypertensive, vasodilating, neuroprotective, antidepressant, antiarrhythmic, antiepileptic, and antitumor activities [1, 2].

In 1975, a combination of thin-layer chromatography, gas-liquid chromatography, ultraviolet spectroscopy, and mass spectrometry techniques for the alkaloid screening of herbarium samples of the genus *Uncaria* (Rubiaceae) was described [9]. Some sixty alkaloids were distinguished by the screening procedure. Serving as the technical base, an increasing number of methods, like high-performance liquid chromatography (HPLC) [10–12], were reported for the determination of *Uncaria* alkaloids. However, the limitations of HPLC include long analysis time and a lack of long-term reproducibility. Liquid chromatography combined with mass spectrometry (LC-MS) is considered a better technique for the simultaneous determination of diversified *Uncaria* alkaloids [13, 14], but these reports had several drawbacks, such as long analysis time (both more than 13 min) and large volume (100 *μ*L) of rat plasma and brain samples, which make them unsuitable for serial blood sampling in mice pharmacokinetic evaluation [13],

UPLC-MS/MS technology is specifically designed for small particle-sized fillers and ultrahigh pressure requirements. It is based on the basic principles of traditional high-performance liquid chromatography systems, but its separation efficiency and the separation speed have been greatly improved, which can significantly improve the resolution and detection sensitivity of chromatographic peaks, especially when combined with mass spectrometry. Wu et al. used the UPLC-MS/MS method, which is faster, more sensitive, and with higher sample throughput compared with LC-MS/MS, to study the pharmacokinetics of rhynchophylline and hirsutine in rat plasma after oral administration. The analysis time is only 6 min, but the sample preparation of rat plasma achieved by alkalization and liquid–liquid extraction is cumbersome because of the long time for evaporation of the extracting solvent and large sample volumes [15]. To our knowledge, the profile of toxicity or pharmacokinetics of some drugs could change in different species. The mouse is the most frequently used species for the preclinical efficacy, toxicology, biodistribution, and pharmacokinetic studies, but there are no published data that demonstrate simultaneous determination of six *Uncaria* alkaloids (corynoxeine, isocorynoxeine, rhynchophylline, isorhynchophylline, hirsutine, and hirsuteine) in mouse blood. Moreover, bioavailabilities of these alkaloids have not been studied yet. Therefore, we standardized and validated a rapid and sensitive UPLC-MS/MS analysis method for simultaneous determination of the concentration of corynoxeine, isocorynoxeine, rhynchophylline, isorhynchophylline, hirsutine, and hirsuteine in mouse blood. This method was successfully applied to the pharmacokinetic and bioavailability study of six *Uncaria* alkaloids in mice after intravenous and gavage administration.

## 2. Materials and Methods

### 2.1. Chemicals and Reagents

**Figure 1:**
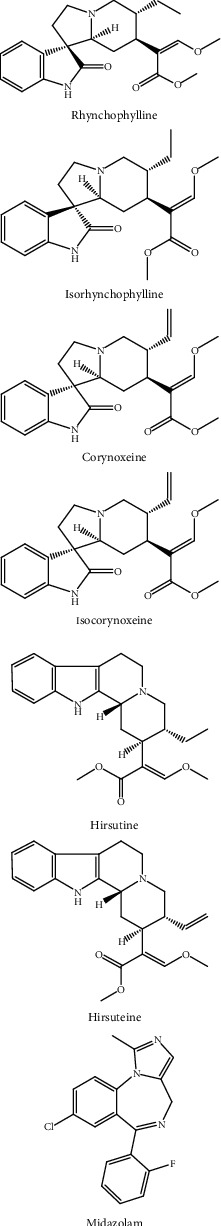
Chemical structure of rhynchophylline, isorhynchophylline, corynoxeine, isocorynoxeine, hirsutine, hirsuteine, and midazolam (IS).

Corynoxeine, isocorynoxeine, rhynchophylline, isorhynchophylline, hirsutine, hirsuteine (all > 98%, Figure 1) and the internal standard midazolam (IS, all > 98%) were purchased from Chengdu Mansite Bio-Technology Co., Ltd. (Chengdu, China). Methanol and acetonitrile (HPLC grade) were purchased from Merck Company (Darmstadt, Germany). Ultrapure water was prepared by a Milli-Q purification system (Millipore, Bedford, USA).

### 2.2. UPLC-MS/MS Conditions

ACQUITY H-Class UPLC and a XEVO TQ-S micro triple quadrupole mass spectrometer equipped with an ESI interface were used in this work (Waters Corp., Milford, MA, USA). Corynoxeine, isocorynoxeine, rhynchophylline, isorhynchophylline, hirsutine, hirsuteine, and IS were separated using a Waters BEH C18 column (2.1 mm × 100 mm, 1.7 *μ*m, Waters, USA), and the column temperature was set at 40°C. The mobile phase consisted of 0.1% formic acid and acetonitrile with a flow rate of 0.4 mL/min. The gradient elution was designed as follows: 0-0.2 min, 10% acetonitrile; 0.2-3.0 min, acetonitrile changed from 10% to 35%; 3.0-3.5 min, 35% acetonitrile; 3.5-4.0 min, acetonitrile descending to 10%; and 4.0-5.5 min, acetonitrile maintained at 10%.

**Figure 2:**
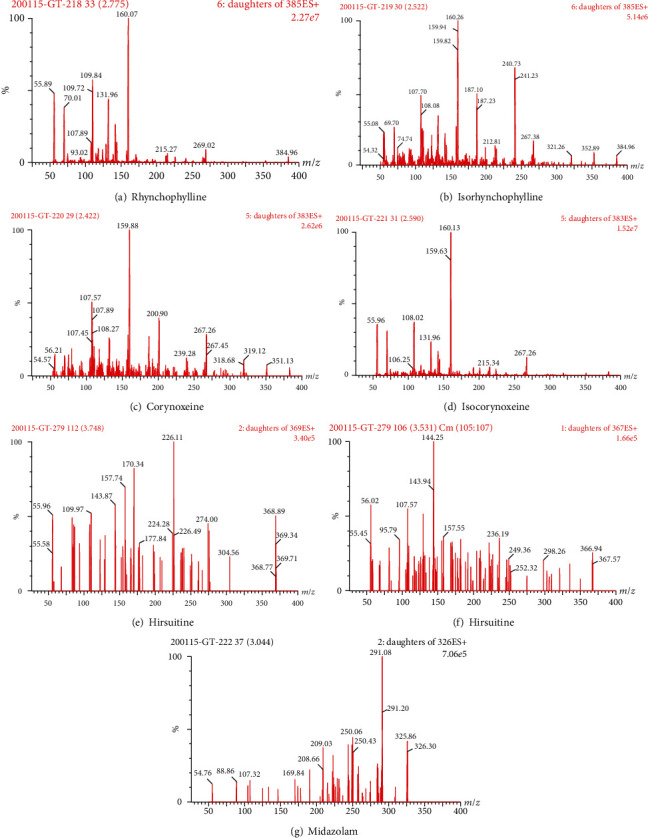
Mass spectrum of rhynchophylline, isorhynchophylline, corynoxeine, isocorynoxeine, hirsutine, hirsuteine, and midazolam (IS).

Desolvation gas (nitrogen) and cone gas (nitrogen) were set at 900 L/h and 50 L/h, respectively. Helium was chosen as the collision gas. Capillary voltage was set at 2.4 kV. The source temperature and desolvation temperature were set at 150°C and 450°C, respectively. Multiple reaction monitoring (MRM) and positive ESI mode were used for quantitative analysis (Table 1, Figure 2).

### 2.3. Preparation of Standard Solutions

Stock solutions (100 *μ*g/mL) of corynoxeine, isocorynoxeine, rhynchophylline, isorhynchophylline, hirsutine, hirsuteine, and IS were prepared in methanol. A series of working solutions were gradually diluted with methanol from the stock solution.

The calibration standards were prepared by spiking blank mouse blood with appropriate amounts of corynoxeine, isocorynoxeine, rhynchophylline, isorhynchophylline, hirsutine, and hirsuteine. Calibration plots of each analyte were constructed in the range of 1-1000 ng/mL with blood (1, 5, 20, 40, 100, 200, 400, and 1000 ng/mL). Quality-control (QC) samples were prepared in three different blood concentrations (4, 90, and 900 ng/mL) in blank mouse blood.

### 2.4. Sample Preparation

In a 0.5 mL centrifuge tube, 100 *μ*L acetonitrile (containing IS 50 ng/mL) was added to the 20 *μ*L blood sample. These tubes were vortex mixed for 0.5 min and centrifuged at 14900 g for 10 min, and then, the supernatant (2 *μ*L) was injected into the UPLC-MS/MS for analysis.

### 2.5. Method Validation

Validation methods were established in accordance with the FDA guidelines for validation of bioanalytical methods. Validation items included selectivity, matrix effects, linearity, precision, accuracy, recovery, and stability [16, 17].

The selectivity of the method was evaluated by analyzing blank samples in the whole blood to minimize or avoid interference from IS and endogenous matrix components. A calibration curve of different concentrations was prepared with standard working solutions of each analyte. Linear regressions of the peak area ratios (*y*) of each analyte to the IS versus the corresponding concentration (*x*) of the analyte were fitted over the quantitation range of 1-1000 ng/mL.

Precision and accuracy were assessed by measuring the QC samples at six repetitions. Precision was expressed as relative standard deviation (RSD), and precision for interday and intraday was determined by measuring the QC samples at three concentration levels in one day and on three consecutive days. The accuracy for intraday and interday was determined by measuring the mean value of QC samples at three concentration levels in accordance with the true value in one day and on three consecutive days.

The recovery rate was evaluated by comparing the measured peak area of the extracted QC samples at low, medium, and high concentrations with the corresponding peak area of extracts of blanks spiked with the analyte standard solution. The matrix effect was evaluated by comparing the peak areas obtained from QC samples at low, medium, and high concentrations with the peak areas obtained from standard solutions of corresponding concentrations.

The stability of corynoxeine, isocorynoxeine, rhynchophylline, isorhynchophylline, hirsutine, and hirsuteine in whole blood of mice was investigated by analyzing the QC samples at low, medium, and high concentrations under the following three storage conditions: short-term stability (2 h at room temperature), long-term stability (-20°C, 30 days), and freeze-thaw (-20°C to room temperature) stability for 3 consecutive days.

### 2.6. Pharmacokinetic Study

Twelve Institute for Cancer Research (ICR) mice (male, 20-22 g) were raised in the Laboratory Animal Center of Wenzhou Medical University. They were divided randomly into two groups, six mice in each group. Alkaloids were precisely weighed and dissolved completely in 0.0017% hydrochloric acid. Then, one group was intravenously administered a mixture of rhynchophylline (1 mg/kg), isorhynchophylline (1 mg/kg), corynoxeine (1 mg/kg), isocorynoxeine (1 mg/kg), hirsutine (1 mg/kg), and hirsuteine (1 mg/kg); and the other group was orally administered a mixture of rhynchophylline (5 mg/kg), isorhynchophylline (5 mg/kg), corynoxeine (5 mg/kg), isocorynoxeine (5 mg/kg), hirsutine (5 mg/kg), and hirsuteine (5 mg/kg).

Blood samples (20 *μ*L) were collected from the tail vein into heparinized 0.5 mL polythene tubes at 0.1667, 0.25, 0.5, 1, 1.5, 2, 3, 4, 6, and 12 h after administration. Pharmacokinetic parameters were analyzed by DAS 2.0 software (China Pharmaceutical University).

## 3. Results

### 3.1. Selectivity

**Figure 3:**
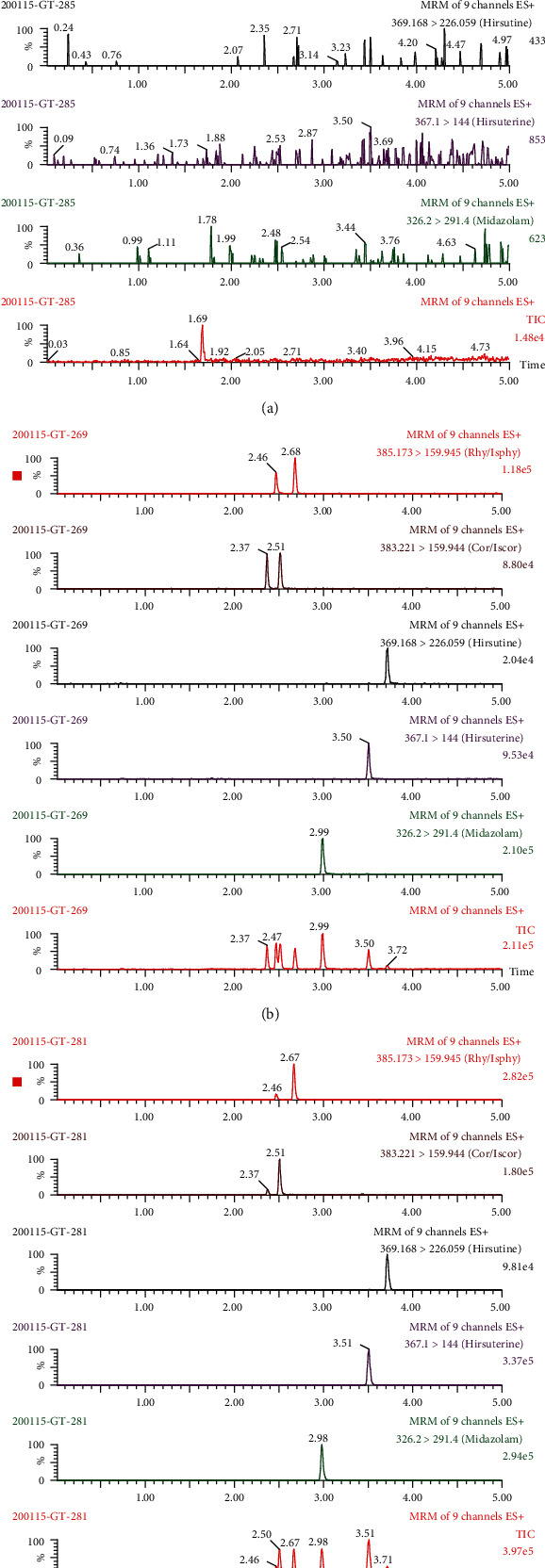
UPLC-MS/MS chromatograms of rhynchophylline, isorhynchophylline, corynoxeine, isocorynoxeine, hirsutine, hirsuteine, and IS in mouse blood. (a) Blank blood; (b) blank blood spiked with rhynchophylline, isorhynchophylline, corynoxeine, isocorynoxeine, hirsutine, hirsuteine, and IS; (c) and a mouse blood after intravenous administration.

Typical chromatograms of blank mouse blood spiked with or without corynoxeine, isocorynoxeine, rhynchophylline, isorhynchophylline, hirsutine, hirsuteine, and IS are shown in Figure 3. No interfering endogenous substances were found at the retention time of the analytes and IS.

### 3.2. Calibration Curve and Sensitivity

Typical equations of the calibration curves for corynoxeine, isocorynoxeine, rhynchophylline, isorhynchophylline, hirsutine and hirsuteine in mouse blood are shown in Table 2. The lower limits of quantifications (LLOQs) were 1 ng/mL.

### 3.3. Precision, Accuracy, Recovery, and Matrix Effect

As shown in Table 3, precision for intraday and interday precision was found to be less than 15%. The accuracy ranged from 86.5% to 110.4%. The recovery ranged between 64.4% and 86.8%. The matrix effects ranged between 94.1% and 109.4%.

### 3.4. Stability

The results of stability indicated that corynoxeine, isocorynoxeine, rhynchophylline, isorhynchophylline, hirsutine, and hirsuteine were stable under various storage conditions (Table 4).

### 3.5. Pharmacokinetics

**Figure 4:**
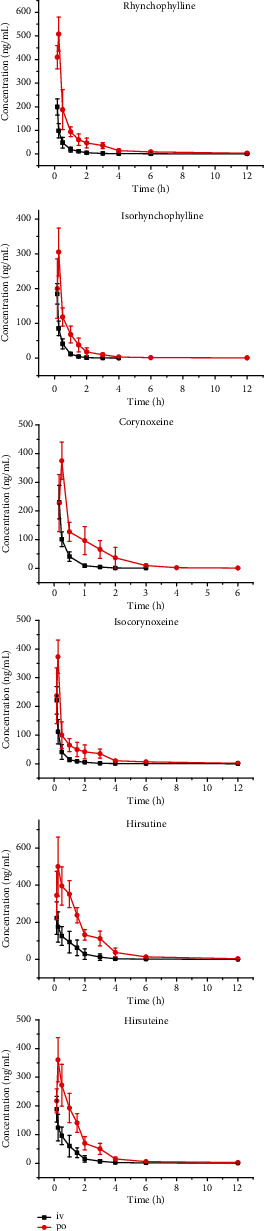
The pharmacokinetics profiles of rhynchophylline, isorhynchophylline, corynoxeine, isocorynoxeine, hirsutine, and hirsuteine in mice (oral 5 mg/kg, intravenous 1 mg/kg) (*n* = 6).

The UPLC-MS/MS method was applied in the pharmacokinetics of corynoxeine, isocorynoxeine, rhynchophylline, isorhynchophylline, hirsutine, and hirsuteine in mice. The main pharmacokinetic parameters of these six alkaloids in the noncompartment model are listed in Table 5. The mean blood concentration-time curves are shown in Figure 4. Bioavailability of corynoxeine, isocorynoxeine, rhynchophylline, isorhynchophylline, hirsutine, and hirsuteine was determined to be 27.3%, 32.7%, 49.4%, 29.5%, 68.9%, and 51.0%, respectively.

## 4. Discussion

In the optimization of mass spectrometry detection methodologies, we examined the sensitivity of the positive and negative electrodes. In this study, the sensitivity of the positive electrode for detection of corynoxeine, isocorynoxeine, rhynchophylline, isorhynchophylline, hirsutine, and hirsuteine was significantly higher than that of the negative electrode for detection of the above alkaloids. We further optimized the ionization mode and found that the MRM mode was more selective and sensitive than the single ion monitoring (SIM) mode. The ionized cone voltage and collision voltage were then optimized. The final results are listed in Table 2. The ion fragments are listed in Figure 2.

The selection result of chromatographic conditions showed that no interfering peaks were found at or near the retention time of the analyte and IS, and the peak shape of the analyte and IS was sharp.

Different combinations of mobile phases were investigated to obtain perfect separation and a better peak shape, including acetonitrile, methanol, formic acid, ammonium acetate, and water. The result showed that acetonitrile-0.1% formic acid had the best sensitivity and peak shape. The gradient elution method was used to improve the peak shape and to avoid matrix effects in this work (Figure 3). Then, different columns, such as HSS C18 (2.1 mm × 100 mm, 1.7 *μ*m), HILIC C18 (2.1 mm × 100 mm, 1.7 *μ*m), BEH C18 (2.1 mm × 100 mm, 1.7 *μ*m), and BEH C18 (2.1 mm × 50 mm, 1.7 *μ*m) were compared, and finally the BEH C18 (2.1 mm × 100 mm, 1.7 *μ*m) column was selected for analyte separation.

The selection of IS is very important in biological detection methods. The position of the chromatographic peak of the IS should be before or in the middle of all chromatographic peaks of the measured analytes. Midazolam was chosen as the IS because of a similar retention time and ionization as corynoxeine, isocorynoxeine, rhynchophylline, isorhynchophylline, hirsutine, and hirsuteine in the positive-ion ESI mode.

An effective and simple sample preparation was the key point for establishing the UPLC-MS/MS method, which included protein precipitation, liquid-liquid extraction, and solid-phase extraction. Finally, the precipitated protein with 5 times the volume of acetonitrile has advantages such as the highest recovery and an acceptable matrix effect.

The developed UPLC-MS/MS method used for corynoxeine, isocorynoxeine, rhynchophylline, isorhynchophylline, hirsutine, and hirsuteine showed that it needed less analysis time than conventional HPLC or LC-MS. It took only 5.5 min for analyzing the whole blood sample. For the first time, the UPLC-MS/MS method was developed for the determination of *Uncaria* alkaloids in the blood of mice, and the relevant methodological process was verified. Methodological verification results showed that the established UPLC-MS/MS method is accurate, fast, highly sensitive, and effective for the detection and pharmacokinetic study of *Uncaria* alkaloids in mouse blood. The pharmacokinetic results showed that the six *Uncaria* alkaloids metabolized rapidly in mice with a half-life between 0.6 h and 4.4 h. The bioavailability of corynoxeine, isocorynoxeine, rhynchophylline, isorhynchophylline, hirsutine, and hirsuteine was 27.3%, 32.7%, 49.4%, 29.5%, 68.9%, and 51.0%, respectively, which showed satisfactory oral absorption of each alkaloid. In our study, the bioavailability of hirsutine and hirsuteine in mice was very different from that in rats, which showed relatively low levels (4.4% and 8.2%, respectively) [18]. This means that some alkaloids are indeed altered in different species.

## 5. Conclusion

A simple, rapid, and selective UPLC–MS/MS method was developed in this work for simultaneous determination of corynoxeine, isocorynoxeine, rhynchophylline, isorhynchophylline, hirsutine, and hirsuteine in mouse blood; it only needed 20 *μ*L blood for sample preparation. Then, the UPLC-MS/MS method was successfully applied in the pharmacokinetics in mice after intravenous and oral administration. The bioavailability of six *Uncaria* alkaloids (ranging between 27.3% and 68.9%) was reported for the first time in mice, which indicates that the most of *Uncaria* alkaloids are easily absorbed into the blood circulatory system through the gastrointestinal tract.

## Figures and Tables

**Table 1:** Mass parameters of rhynchophylline, isorhynchophylline, corynoxeine, isocorynoxeine, hirsutine, hirsuteine, and midazolam (IS).

Compound name	Parent (*m*/*z*)	Daughter (*m*/*z*)	Cone (V)	Collision (V)	Retention time (min)
Rhynchophylline	385.2	159.9	36	34	2.68
Isorhynchophylline	385.2	159.9	36	34	2.46
Corynoxeine	383.2	159.9	42	30	2.37
Isocorynoxeine	383.2	159.9	42	30	2.51
Hirsutine	369.2	226.1	30	24	3.75
Hirsuteine	367.1	144.0	30	30	3.50
Midazolam (IS)	326.2	291.4	30	25	2.99

**Table 2:** Regression equation and correlation coefficient for rhynchophylline, isorhynchophylline, corynoxeine, isocorynoxeine, hirsutine, and hirsuteine in mouse blood (*y* = peak area ratio of the analyte versus IS; *x* = concentration of the analyte).

Compound	Linear range (ng/mL)	Regression equation	Correlation coefficient
Rhynchophylline	1-1000	*y* = 0.0082*x* − 0.0021	0.9988
Isorhynchophylline	1-1000	*y* = 0.0043*x* + 0.0021	0.9972
Corynoxeine	1-1000	*y* = 0.0049*x* + 0.0002	0.9990
Isocorynoxeine	1-1000	*y* = 0.0131*x* − 0.0125	0.9992
Hirsutine	1-1000	*y* = 0.0019*x* + 0.0012	0.9976
Hirsuteine	1-1000	*y* = 0.0016*x* − 0.0010	0.9960

**Table 3:** Precision, accuracy, extraction efficiency, and matrix effect of rhynchophylline, isorhynchophylline, corynoxeine, isocorynoxeine, hirsutine, and hirsuteine in mouse blood (*n* = 6).

Compound	Concentration (ng/mL)	Accuracy (%)		Precision RSD (%)		Matrix Effect	Recovery
		Intraday	Interday	Intraday	Interday		
Rhynchophylline	1	105.2	108.4	12.0	14.1	106.7	68.7
	4	101.6	93.4	9.1	10.1	108.4	67.6
	90	97.1	106.6	10.6	7.4	103.6	64.6
	900	103.0	98.6	4.9	8.7	102.0	72.0
Isorhynchophylline	1	108.8	109.0	14.0	11.1	94.1	71.0
	4	97.6	90.6	11.6	6.8	103.3	83.5
	90	98.8	109.8	4.7	7.5	100.8	69.5
	900	102.9	96.3	5.1	2.5	95.6	79.5
Corynoxeine	1	97.8	90.7	11.3	13.3	106.1	75.9
	4	100.7	97.6	12.5	8.9	105.6	69.6
	90	99.0	104.6	5.1	12.2	107.4	73.9
	900	95.5	96.3	6.8	4.5	106.6	81.3
Isocorynoxeine	1	86.5	110.4	9.5	12.1	108.4	79.2
	4	97.0	92.9	11.4	11.7	105.9	86.8
	90	107.4	93.1	9.0	12.3	106.1	81.4
	900	101.3	100.2	7.2	6.4	104.0	80.9
Hirsutine	1	107.6	92.7	11.9	14.1	103.9	78.8
	4	97.1	97.0	6.1	13.5	102.4	72.6
	90	107.0	96.0	5.9	10.5	109.4	70.3
	900	104.3	97.8	5.0	9.0	107.8	76.4
Hirsuteine	1	98.7	109.0	13.6	12.0	103.6	73.3
	4	108.1	97.3	7.9	8.5	105.1	64.4
	90	102.1	94.7	8.6	10.2	105.6	76.1
	900	100.2	105.9	10.0	3.2	108.2	65.0

**Table 4:** Stability of rhynchophylline, isorhynchophylline, corynoxeine, isocorynoxeine, hirsutine, and hirsuteine in mouse blood.

Compound	Concentration (ng/mL)	Autosampler ambient		Ambient 2 h		-20°C 30 d		Freeze-thaw	
		Accuracy	RSD	Accuracy	RSD	Accuracy	RSD	Accuracy	RSD
Rhynchophylline	4	98.8	6.7	102.6	7.3	87.2	11.2	81.4	11.1
	90	106.7	2.3	101.1	1.4	103.4	9.5	104.4	9.3
	900	105.4	6.0	95.0	8.2	98.3	8.6	96.2	10.7
Isorhynchophylline	4	105.6	7.1	97.5	11.4	99.7	9.2	106.4	8.0
	90	103.7	8.2	108.9	8.2	107.1	13.5	104.4	12.1
	900	101.4	4.6	93.5	6.9	89.4	4.8	105.5	9.9
Corynoxeine	4	96.8	5.5	103.9	2.4	96.1	11.3	91.5	13.9
	90	97.6	2.8	101.5	6.5	101.8	5.5	98.1	7.0
	900	101.7	4.1	105.2	5.7	107.9	2.8	105.7	9.0
Isocorynoxeine	4	103.5	6.4	106.0	8.9	102.9	7.5	107.7	9.7
	90	97.2	6.4	97.2	4.5	96.5	7.2	95.0	11.5
	900	103.1	3.7	98.7	3.9	101.5	8.9	107.7	6.6
Hirsutine	4	101.9	10.6	95.3	12.9	111.6	11.2	92.0	12.1
	90	97.2	8.2	103.9	11.7	95.9	7.7	103.2	11.0
	900	100.2	8.5	102.3	4.8	110.6	11.4	92.3	12.1
Hirsuteine	4	96.6	9.3	95.1	11.3	99.0	12.6	109.8	13.6
	90	99.6	7.8	107.5	9.0	93.0	11.4	92.0	10.2
	900	100.1	5.1	96.4	5.9	103.2	11.9	99.8	9.9

**Table 5:** Pharmacokinetic parameters of rhynchophylline, isorhynchophylline, corynoxeine, isocorynoxeine, hirsutine, and hirsuteine after oral (po) and intravenous (iv) administration in mice (mean ± SD, *n* = 8).

Parameters	Unit	Rhynchophylline		Isorhynchophylline		Corynoxeine		Isocorynoxeine		Hirsutine		Hirsuteine	
		iv	po	iv	po	iv	po	iv	po	iv	po	iv	po
AUC_(0 − *t*)_	ng/mL^∗^h	168.6 ± 41.8	416.7 ± 82.9	142.0 ± 37.2	209.2 ± 48.1	191.4 ± 99.7	260.9 ± 66.7	179.2 ± 67.3	293.1 ± 95.7	261.3 ± 122.8	899.7 ± 157.8	200.7 ± 49.8	511.3 ± 83.3
AUC_(0-∞)_	ng/mL^∗^h	169.4 ± 41.6	459.6 ± 122.7	142.2 ± 37.2	212.1 ± 51.5	191.8 ± 99.7	261.8 ± 67.1	180.4 ± 66.5	299.1 ± 91.1	261.7 ± 122.5	908.4 ± 158.3	202.4 ± 49.5	518.2 ± 81.1
MRT_(0 − *t*)_	h	0.5 ± 0.2	1.7 ± 0.6	0.2 ± 0.1	1.2 ± 0.1	0.2 ± 0.1	0.9 ± 0.2	0.3 ± 0.1	1.6 ± 0.4	0.9 ± 0.4	1.8 ± 0.3	0.9 ± 0.3	1.6 ± 0.3
MRT_(0-∞)_	h	0.6 ± 0.4	3.8 ± 4.9	0.2 ± 0.1	1.4 ± 0.4	0.2 ± 0.1	0.9 ± 0.2	0.5 ± 0.3	1.8 ± 0.2	1.0 ± 0.4	1.9 ± 0.5	1.0 ± 0.5	1.9 ± 0.5
*t*_1/2z_	h	2.2 ± 1.4	4.4 ± 6.0	0.6 ± 0.3	2.5 ± 2.1	0.5 ± 0.2	0.7 ± 0.4	3.2 ± 2.7	1.6 ± 0.7	1.1 ± 0.6	2.0 ± 0.9	2.2 ± 1.4	2.4 ± 2.0
Vz	L/kg	20.0 ± 13.4	60.1 ± 67.1	6.8 ± 4.0	84.5 ± 57.5	4.4 ± 1.5	19.3 ± 9.1	28.7 ± 26.2	45.7 ± 36.1	8.3 ± 6.5	16.8 ± 9.0	15.9 ± 9.8	36.1 ± 33.2
CL_*z*/*F*_	L/h/kg	6.2 ± 1.5	11.6 ± 3.2	7.4 ± 1.8	24.7 ± 6.1	6.2 ± 2.3	20.2 ± 5.2	6.1 ± 1.9	18.2 ± 6.1	4.4 ± 1.7	5.6 ± 1.0	5.2 ± 1.5	9.8 ± 1.5
*C*_max_	ng/mL	199.3 ± 34.3	508.0 ± 71.9	184.6 ± 29.3	305.3 ± 68.8	230.7 ± 58.1	374.8 ± 65.1	221.2 ± 47.8	373.2 ± 58.3	222.6 ± 87.6	524.5 ± 124.5	188.4 ± 44.7	360.8 ± 77.2
Bioavailability			49.4%		29.5%		27.3%		32.7%		68.9%		51.0%

## Data Availability

The data used to support the findings of this study are included within the article.
